# Molecular structure of starch isolated from jackfruit and its relationship with physicochemical properties

**DOI:** 10.1038/s41598-017-13435-8

**Published:** 2017-10-18

**Authors:** Yanjun Zhang, Yutong Zhang, Fei Xu, Gang Wu, Lehe Tan

**Affiliations:** 1 Spice and Beverage Research Institute, Chinese Academy of Tropical Agricultural Sciences, Wanning, 571533 Hainan China; 2 College of Food Science, Bayi Agricultural University, Daqing, Heilongjiang 158308 China

## Abstract

The molecular structure of starches isolated from five jackfruits (M2, M3, M4, M8 and X1) and its relationship with physicochemical properties were investigated. Although they had uniform amylose (AM) content, the five jackfruit starches displayed different physicochemical properties, including their pasting, thermal, crystal and texture properties. Furthermore, differences in the molecular structure (i.e., average weight-average molar mass (Mw) of amylose and amylopectin (AP) as well as the same AP fine structure) were also found in the five jackfruit starches. The results indicated that jackfruit starch with a larger Mw of amylose and proportions of DP 25–36, DP ≥ 37 and chain length had a lower peak viscosity, breakdown, final viscosity, setback and adhesiveness, but a higher pasting and gelatinization temperature, gelatinization temperature range, gelatinization enthalpy and relative crystallinity. *Xiangyinsuo 1 hao* (X1) starch, which originated from Xinglong in Hainan province, China, had special physicochemical properties, which were ascribed to its lower amylopectin Mw, smaller particle size, and perfect amylopectin structure. The results showed that the most important intrinsic factors that could determine the physicochemical properties of starch were its molecular structure, including the Mw of amylose and AP as well as a fine AP structure.

## Introduction

Jackfruit (*Artocarpus heterophyllus Lam*) is one of the most common evergreen trees found in tropical and subtropical regions of the world and is widely cultivated for its large edible fruit. The varieties grown in China include some originating from *Malaysia*, such as *Malaysia No. 1–6*, as well as local varieties such as *XingLongBenDi* ^1^. Jackfruit is currently cultivated in the provinces of Hainan, Guangdong, Guangxi and Yunnan, and the total plantation area is more than 15,000 hectares. Jackfruit is composed of rind, edible yellow flesh, procarp and seeds. Jackfruit seeds which represent 8–15% of the fruit weight^2^ have a short shelf life because of their high carbohydrate content (i.e., starch content above 60%, dry basis) and could thus be used as good culture for microorganism^3^. Although they contain a large amount of potentially useful starchy material, the seeds are usually discarded as a waste in jackfruit processing, thus contributing to environmental pollution^4^. Due to their high starch concentration, the best way to preserve the seeds is to extract the starch, which is used in different industries based on its suitability.

There are a few studies on the physicochemical properties of a variety of jackfruit, mainly concerning seed composition, starch chemical composition, swelling power and solubility, pasting, gelatinization, crystallization and digestibility properties. Tulyathan *et al*. investigated the physicochemical properties of the “Thong Sud Jai” jackfruit starch in terms of its seed composition, starch chemical composition, pasting, and crystallization properties^5^. Madruga *et al*. studied the starch chemical composition, swelling power and solubility, morphological, and gelatinization properties for the soft and hard jackfruit varieties^6^. Phrukwiwattanakul, Wichienchotand and Sirivongpaisal studied the starch chemical composition, pasting and gelatinization properties of “Thong Prasert” jackfruit from Thailand^7^. Kittipongpatana and Kittipongpatana investigated the starch chemical composition, gelatinization and crystallization properties for “Thong Prasert” from Thailand^8^. Chen *et al*. studied the starch chemical composition, microstructure, gelatinization, crystallization and digestibility properties of Chinese jackfruit. In our previous work, we studied the physicochemical properties and microstructure between different species^9^. However, the molecular structure (i.e., average weight-average molar mass (Mw) of amylose and amylopectin, and the amylopectin fine structure) of jackfruit starches is still unknown. In addition, the differences in the molecular structures between different jackfruit species are unclear. Furthermore, the relationship is not clear between the physicochemical properties and the molecular structure for the different jackfruit species.

The amylopectin (AP) fine structure is of great interest to food scientists and technologists since it profoundly affects the physicochemical and functional properties of starch, including the branch chain length distribution and chain length (CL)^10^. Jane *et al*.^11^ indicated that starches with a short average amylopectin branch chain length had a low gelatinization temperature. Pinhão starches contain amylopectin with a larger proportion of medium-short branch-chains (degree of polymerization, DP, 13–24) and an average branched chain-length of 19.7–21.4 anhydrous glucose units^12^. It was reported that a smaller-sized AP with a higher average external chain length (ECL) and a higher proportion of DP >37 had a higher final viscosity (FV) of the starch^10^. Compared to AP fine structure studies, there are few reports on the molar masses of amylose and amylopectin separated from starch^13,14^. The various average weight-average molar mass (Mw) of amylose (AM) and amylopectin and the corresponding branch chain-length distribution of amylopectin from the same AP sample are still unknown. To the best of our knowledge, the relationship between the physicochemical properties and the molecular structure of amylopectin, including the molecular weight distributions and branch chain-length distribution, is still unclear.

Five new jackfruit species [*Malaysia No. 2 (M2)*, *Malaysia No. 3 (M3)*, *Malaysia No. 4 (M4)*, *Malaysia No. 8 (M8)*, and *Xiangyinsuo 1 hao (X1)*] from China are used as raw materials in this study, which aims to investigate their molecular structure and the physicochemical properties of AP and AM. In addition, a relationship between the molecular structure [average weight-average molar mass (Mw), number-average molar mass (Mn), average radius of gyration (Rg) from amylose and amylopectin and corresponding branch chain-length distribution of amylopectin from the same AP sample] and the physicochemical properties (pasting, thermal, crystal and textural properties) is preliminarily studied.

## Results

### Morphology, size distribution and amylose content

The starch particle size distributions are shown in Table S1. The sizes of the five different samples ranked as M3 > M2 > M4 > M8 > X1. Among the starches, M3 showed the widest range of 0.46–46.95 μm and the largest particle size, with an average of 12.46 μm, whereas X1 starch showed the narrowest range of 0.41–30.77 μm and the smallest, with an average size of 7.63 μm. Significant variations in the average size were observed in starch granules from different cultivars (p < 0.05, Table 1). The amylose content of the jackfruit seed starches was in the range of 30.05–32.56 g/100 g starch and showed no significant differences among the five starches (p < 0.05) (Table S1).

**Table 1:** Mw, Mn, Rg and PI of amylose and amylopectin measured by HPSEC-MALLS-RI.

Starch	Amylose				Amylopectin			
	Mw (10^6^ g/mol)	Mn (10^6^ g/mol)	Rg (nm)	PI	Mw (10^7^ g/mol)	Mn (10^7^ g/mol)	Rg (nm)	PI
X1	2.61b	1.09b	72.1b	2.39a	3.61c	2.98c	94.6b	1.21a
M8	3.79a	1.55a	79.1a	2.45a	2.51c	1.87c	95.8b	1.35a
M4	2.12c	0.92b	61.6c	2.30a	6.45a	4.57a	132.2a	1.41a
M3	2.03c	0.84c	59.5c	2.42a	6.74a	5.36a	136.5a	1.26a
M2	2.57b	0.99b	71.8b	2.59a	4.64b	3.48b	126.2a	1.33a

Values followed by the same letter in the same column are not significantly different (P < 0.05).

## Molecular Structure

### Molecular weight distribution

**Figure 1:**
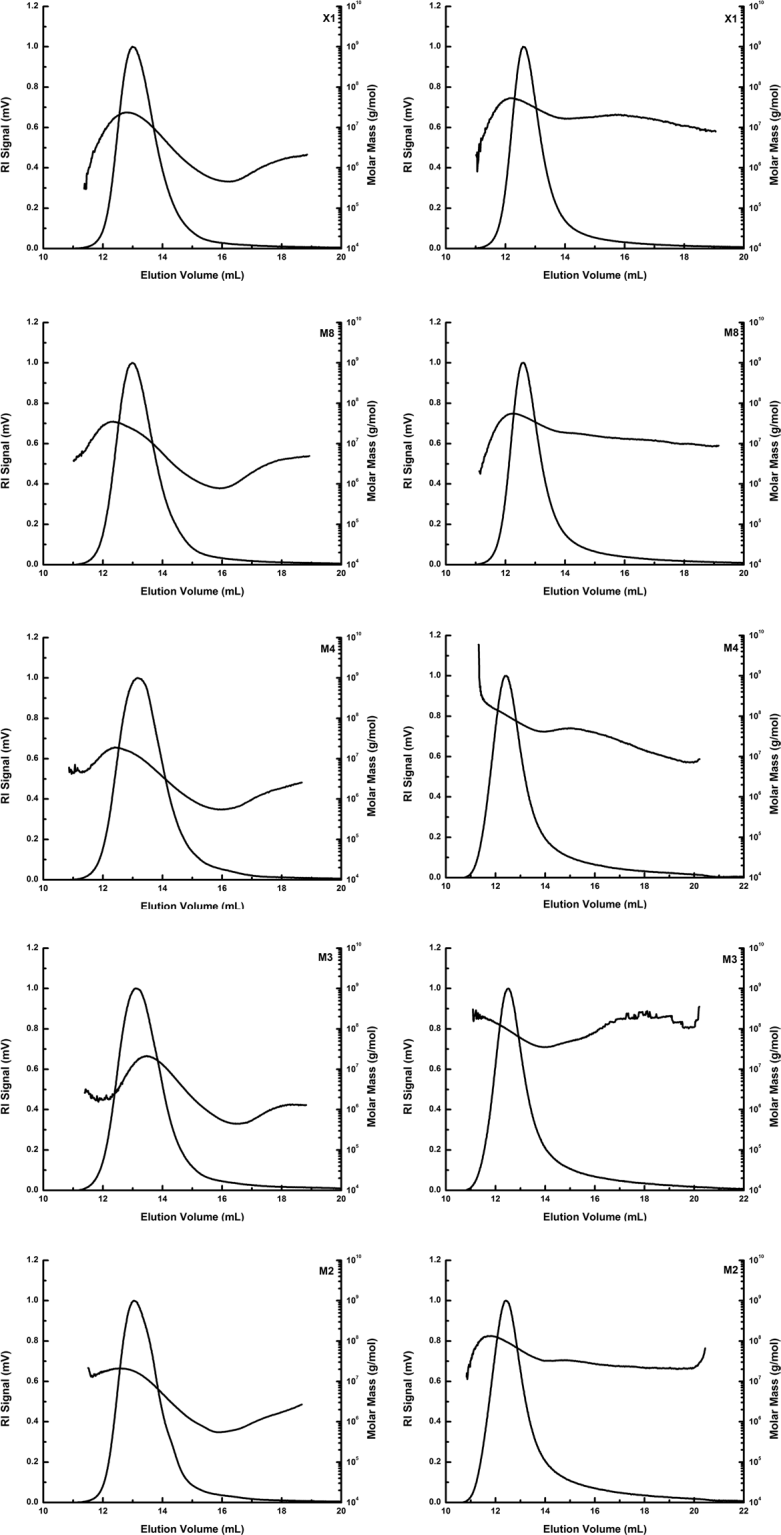
Elution volume vs. molar mass of amylose (left) and amylopectin (right) for starches of five jackfruit varieties.

The molecular weight distribution of amylose and amylopectin of the five starches eluted by the mobile phase DMSO/NaNO_3_ and traced by multi-angle laser light scattering (MALLS) and a refractive index (RI) detector are shown in Fig. 1. Table 1 shows the weight-average molar mass (Mw), the number-average molar mass (Mn), the average radius of gyration (Rg) and the PI (Mw/Mn) of amylose and amylopectin calculated based on the Berry fit model. Significant differences (p < 0.05) were observed for Mw, Mn, and Rg of amylose and amylopectin (Table 1).

M3 amylose had the smallest Mw, Mn and Rg (2.03 × 10^6^ g/mol, 0.84 × 10^6^ g/mol and 59.5 nm), but M8 had the largest, ranging from 2.03–3.79 × 10^6^ g/mol, 0.84–1.55 × 10^6^ g/mol and 59.5–79.1 nm, respectively. Table 1 and Fig. 1A show that no significant differences are observed for the amylose PI among the five samples. The Mw and Rg of the jackfruit starch amyloses were comparable to those of maize, amylomaize and barley starches [1.4–3.1 × 10^6^ g/mol (15–164 nm), 1.5–2.5 × 10^6^ g/mol (60–83 nm), 4.4–5.7 × 10^6^ g/mol (98–107 nm), respectively]^15,16^, which were higher than the values for rice starches (0.28–0.42 × 10^6^ g/mol) but lower than those for potato starch (19.6 × 10^6^ g/mol)^13,17,18^.

As shown in Table 1, the Mw, Mn, Rg and PI of the jackfruit seed amylopectin were in the range of 2.51–6.74 × 10^7^ g/mol, 1.87–5.36 × 10^7^ g/mol, 94.6–136.5 nm, and 1.26–1.41, respectively. Samples M8 and X1 showed significantly (p < 0.05) lower Mw, Mn and Rg than did the other amylopectin samples (Table 1 and Fig. 1B). The PI of amylopectin in the analysed samples did not differ significantly (p < 0.05), which indicated that the amylopectin from five jackfruit cultivars had the same polydispersity. Compared to previous study on Mw and Rg, higher values were reported for maize (7.1–24.3 × 10^7^ g/mol, 194–247 nm), amylomaize (19.7 × 10^7^ g/mol, 214 nm), and barley starches (22.6–28.4 × 10^7^ g/mol, 223–240 nm), and relatively similar values were reported for tapioca (7 × 10^7^ g/mol, 191 nm), and rice starches (5.92–8.80 × 10^7^ g/mol, 134.8–187.2 nm)^10,13,16^.

### Branch chain-length distribution of amylopectin

**Figure 2:**
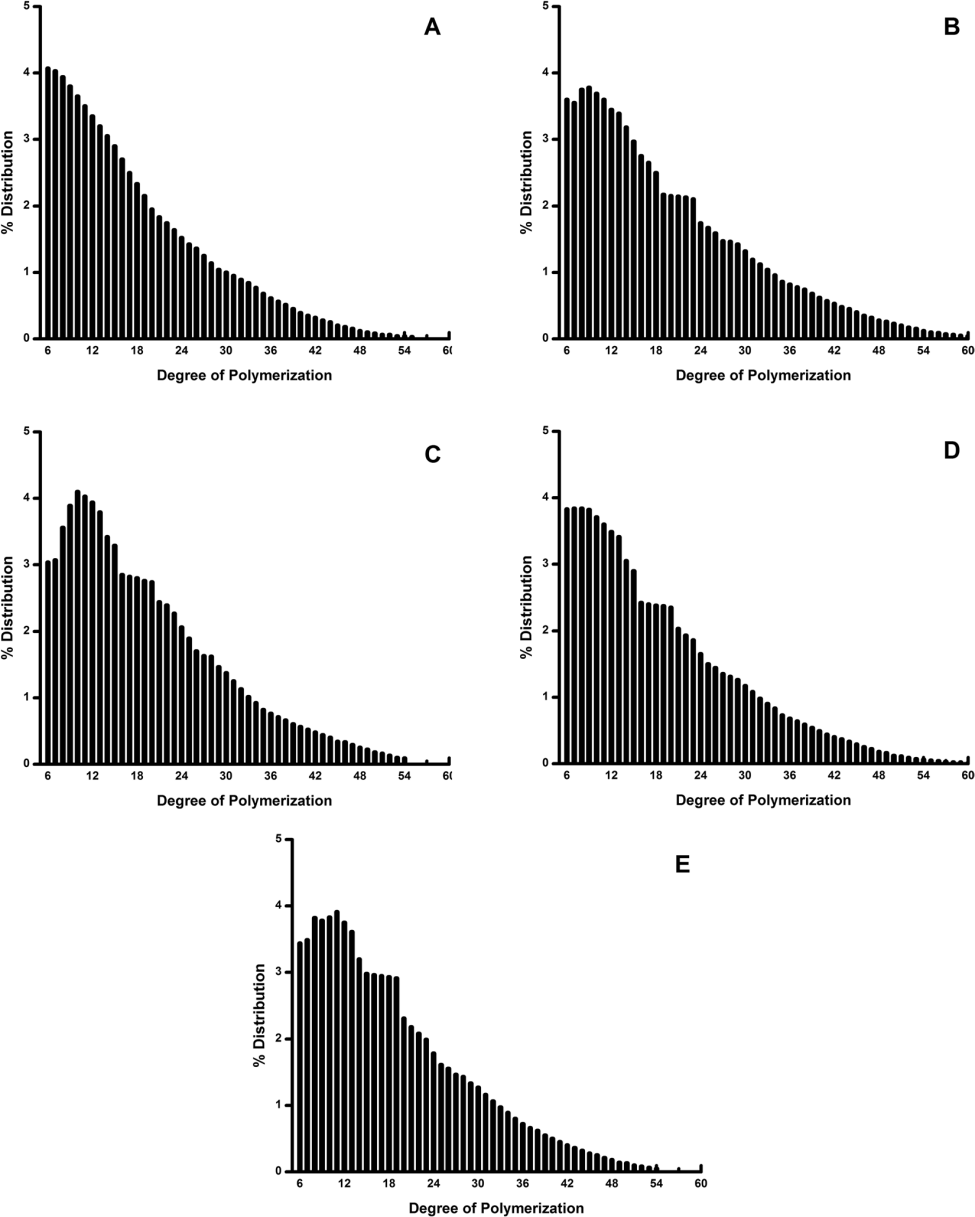
Amylopectin branch chain-length distributions determined by using a high-performance anion-exchange chromatography system equipped with a pulsed amperometric detector (HPAEC-PAD), (**A**) X1, (**B**) M8, (**C**) M4, (**D**) M3, (**E**) M2.

Figure 2 presents the branch chain length distribution of amylopectin obtained via high performance anion exchange chromatography (HPAEC) with a pulsed amperometric detector (PAD) (i.e., a HPAEC-PAD chromatogram). The results are grouped as A chains (DP 6–12), B1 chains (DP 13–24), B2 chains (DP 25–36), and B3 + chains (DP ≥ 37) and are summarized in Table 2. Jackfruit starches from different cultivars exhibited different branch chain length distributions of amylopectin (p < 0.05). The proportions of A chains (DP 6–12), B1 chains (DP 13–24), B2 chains (DP 25–36), and B3 + chains (DP ≥ 37) were 30.34–40.33%, 38.71–41.50%, 14.29–20.98%, and 5.80–9.97%, respectively (Table 2). The average chain length (CL) of jackfruit starches ranged from DP 15.59 to DP 21.22. Starch X1, which originated from Xinglong, Hainan province, China, had the highest proportion of DP 6–12, the smallest proportion of DP 25–36, DP ≥ 37, and the shortest CL (Fig. 2, Table 2), while starch M8 had the smallest proportion of DP 6–12, the highest proportion of DP 25–36, DP ≥ 37, and the longest CL (Fig. 2A,B, Table 2). A shoulder at DP 15–22 was observed for all amylopectin samples except X1. All the jackfruit starches had a higher proportion of DP 6–12 but a lower proportion of DP 25–36, DP ≥ 37 and a shorter CL than found in a previous study of maize starch (21.75%, 14.9%, 19.3%, 24.4), rice starch (23.10%, 12.3%, 16.5%, 22.7), barley starch (25.72%, 17.7%, 12.6%, 22.1) and tapioca starch (21.98%, 15.6%, 26.7%, 27.6)^11^.

**Table 2:** Branch chain-length distributions of jackfruit amylopectin from five cultivars.

Amylopectin	% Distribution				Average CL (DP)
	A(DP 6–12)	B1(DP 13–24)	B2(DP 25–36)	B3(DP ≥ 37)	
X1	40.33a	39.58a	14.29c	5.80c	15.59c
M8	30.34d	38.71a	20.98a	9.97a	21.22a
M4	31.80c	41.50a	18.87b	7.83b	18.34b
M3	35.34b	39.29a	17.89b	7.48b	19.45b
M2	33.93b	40.53a	18.58b	6.96b	18.86b

Values followed by the same letter in the same column are not significantly different (P < 0.05).

According to the characterizations of both the molecular weight distributions and branch chain-length distributions of amylopectin, the five jackfruit seed starches can be divided into three groups: M8; X1; M2, M3, and M4.

### Pasting properties

**Figure 3:**
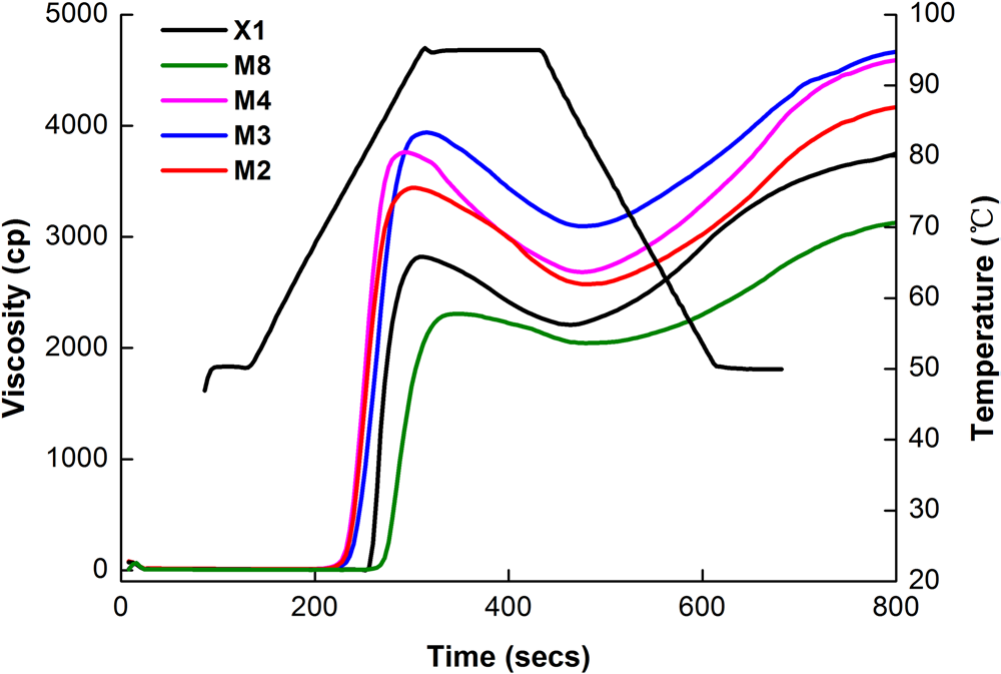
Pasting profiles of seed starches isolated from five different jackfruits cultivars.

The pasting properties of starches from the five jackfruit species are presented in Fig. 3, and their pasting parameters are given in Table 3. The peak viscosity (PV) of the starches varied from 2210 to 3940 cp and was lowest for M8, followed by X1 (Table 3). A previous study indicated that the lower the Mw of amylopectin was, the lower paste viscosity it showed^10^. The PV differences in this study could be due to the lower Mw, and Rg of amylopectin from M8 compared to M2, M3, and M4. In addition, starch M8 had the smallest proportion of DP 6–12 and the highest proportion of DP 25–36, DP ≥ 37, and CL as Table 3 and Fig. 2B show. It was reported that a higher proportion of very long amylopectin branch chains and a longer average amylopectin branch chains lead to a lower PV of starch^12^. StarchX1 had a lower proportion of very long amylopectin branch chains and CL (15.59) and was expected to have a higher PV, but it was found to be lower PV for X1. Based on previous reports^9,19^, this result could be due to a lower Mw of amylopectin and particle size of X1 (Tables 1 and S1, Fig. 1B).

**Table 3:** Pasting properties of seed starches isolated from five different jackfruits cultivars.

Parameters	Peak Viscosity (cp)	Trough Viscosity (cp)	Breakdown	Final Viscosity (cp)	Setback	Pasting Temperature (°C)
X1	2834c	2291c	543c	3768b	1477d	88.88a
M8	2210d	2043d	167d	3055c	979e	91.42a
M4	3763ab	2684b	1079a	4562a	1878a	82.30c
M3	3940a	3097a	843b	4665a	1568c	83.20c
M2	3443b	2575b	868b	4241a	1666b	82.30c

Values followed by the same letter in the same column are not significantly different (P < 0.05).

The breakdown viscosity (BDV) varied significantly from 167 to 1079 cp in jackfruit starches M8 and M4, respectively. The lower BDV observed for starches M8 and X1 suggested its higher shear resistance compared to the group containing M2, M3, M4 and indicated strong cohesive forces within the starch granules and a high thermal stability^12,20^. With the longest CL, the very long branch-chains of amylopectin from the M8 can mimic amylose to form helical complexes with few lipids and can intertwine with other branch chains to maintain the integrity of starch granules during heating and shearing, according to previous reports^11^.

The final viscosity (FV) of the jackfruit starches ranged from 3055 cp to 4665 cp (Table 3 and Fig. 3). The group of M2, M3 and M4 had higher FVs than did M8 and X1. The FV indicates the re-association of the amylose molecules during the cooling period after gelatinization to form a gel network^21^. The differences of FV in the five jackfruits could be due to variations in the amylose weights (Table 1 and Fig. 1A). Based on previous results^22,23^, when amylose was used as a skeleton for amylopectin gelatinization, the higher the Mw of amylose was, the less the gel network formed.

Starches of M2, M3 and M4 showed a higher retrogradation tendency due to their higher setback viscosity.

Among the five cultivars, the pasting temperature of the jackfruit starches varied significantly from 82.30 °C to 91.42 °C (p < 0.05, Fig. 3 and Table 3). M8 and X1 showed higher pasting temperatures than did the group containing M2, M3 and M4. This could have occurred because M8 had the highest proportion of DP 25–36, DP ≥ 37, and the longest CL but the smallest proportion of DP 6–12 (Table 2 and Fig. 2B), as a previous study indicated that the pasting temperature correlated positively with the proportion of very long amylopectin branch chains and long CL^11,20^. According to previous results^11,20^, X1 should be expected to have a lower pasting temperature, with the lowest proportion of very long amylopectin branch chains and the shortest CL (15.59), but the opposite result was observed in this study. If a shoulder was found in the amylopectin chromatogram, it indicated that the short chains formed defective crystallites^11^. The fact that X1 did not show a shoulder in the chromatogram suggested a perfect crystalline structure, which could have contributed to the very high pasting temperature (Fig. 3). It was reported that the pasting temperature was dependent on the starch particle size and that the larger the particles were, the less resistant to rupture and a loss of molecular order they were^9,24^. The higher pasting temperature of X1 might also be explained by its smaller particle size of 7.63 μm (Table S1).

### Thermal properties

**Figure 4:**
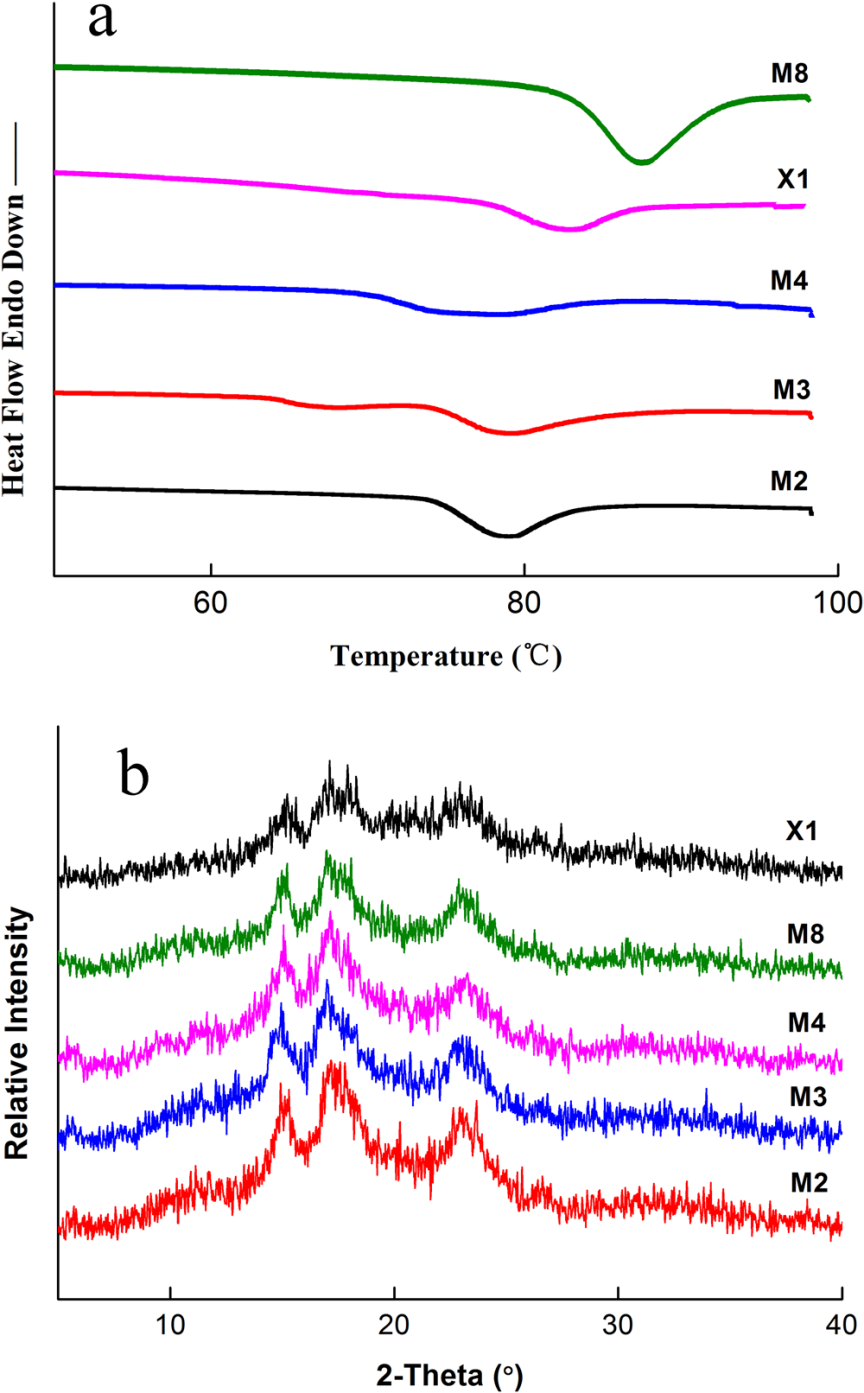
(**a**) DSC curves and (**b**) X-ray diffraction patterns of seed starches isolated from five different jackfruits cultivars.

The thermal properties of the jackfruit starches are summarized in Table 4, and DSC thermographs of the five jackfruit starches are shown in Fig. 4a. The onset gelatinization temperature (To), peak temperature (Tp), conclusion temperature (Tc), gelatinization temperature range (R) and enthalpy of gelatinization (ΔHg) differed significantly (p < 0.05). The transition temperatures (To, Tp and Tc), R and ΔHg ranged from 70.49 to 84.91 °C, 77.26 to 87.43 °C, 88.32 to 96.58 °C, 11.67 to 17.83 °C and 9.14 to 16.78 J/g, respectively (Table 4). M8 and X1 had higher onset and conclusion temperatures than did the group containing M2, M3 and M4. This was ascribed to the fact that the M8 had the longest average branch chain length (21.22) and the largest proportion of long chains (DP 25–36, ≥37, 20.98%, 9.97%, respectively). X1 had a smaller particle size (7.63 μm, Table S1) and a more perfect crystalline structure (Fig. 2) than did the other starches. The DSC results agreed well with the results obtained from the Rapid Visco Analyzer (RVA) data.

**Table 4:** Thermal properties and relative crystallinity of five jackfruit seed starches.

**Parameters**	**To (**°C**)**	**Tp (**°C**)**	**Tc (**°C**)**	**ΔHg (J/g)**	**R (**°C**)**	**Relative crystallinity (%)**
**X1**	77.88b	82.50b	90.32b	9.14e	12.44d	27.34d
M8	84.91a	87.43a	96.58a	16.78a	11.67e	33.95c
**M4**	70.49d	77.26d	88.32c	15.28bc	17.83a	35.21a
**M3**	73.89c	78.84c	90.39b	14.78c	16.50b	34.01b
**M2**	74.43c	78.75c	90.09b	13.91d	15.66c	33.54c

To, onset temperature; Tp, peak temperature; Tc, conclusion temperature; R, gelatinization temperature range; ∆Hg, enthalpy of gelatinization (J/g dry starch). Values followed by the same letter in the same column are not significantly different (P < 0.05).

The enthalpy value (ΔHg) reflects the loss of the ordered double helices^25^, indicating that the five starches had significantly different double helix arrangements (p < 0.05, Table 4). The lowest and highest ΔHg were observed for X1 and M8, respectively (Table 4). The higher ΔHg of M8 suggested a higher percentage crystallinity of the amylopectin, which indicated that more energy is required to break the intermolecular bonds in longer chain lengths of M8 to achieve gelatinization.

### Crystal properties

The XRD of jackfruit starches from different cultivars are presented in Fig. 4B, and the relative crystallinity (RC) data are summarized in Table 4. A doublet with peaks at 15.1°, 17°, 18° and 23.0° was clear for all X-ray diffractograms. These diffractions were recognized as an A-type crystallinity pattern, which corresponded to a close packing of amylopectin double helices^20,22,25,26^. As Table 4 shows, the relative crystallinity ranged from 27.34% to 35.21%, and X1 had the lowest value. As Table 1 and Fig. 2A show, X1 showed the smallest particle size and the smallest proportions of very long amylopectin branch chains and CL, but it displayed a perfect amylopectin shoulder structure and the highest proportions of short amylopectin branch chains. According to previous results^22,26^, this would lead to the formation of the most perfect but the smallest amount of crystalline structure for X1 compared to the other starch samples. Furthermore, X1 had a better orientation of the double helices within the crystalline lamella when it was examined under an X-ray beam. This was also demonstrated by the pasting and gelatinization temperatures reflected by the RVA and DSC results (Tables 3 and 4 and Figs 3 and 4).

### Textural properties

The textural profile analysis (TPA) parameters of five jackfruit seed starches are shown in Table S2. The textural parameters of the starch gels from the various jackfruit varieties varied significantly [probability (p) < 0.05]. The ranges of hardness, adhesiveness, springiness, gumminess, chewiness, resilience and cohesiveness ranged from 529 g, −152.63 g·s, 138.26%, 309.68 g, 150.96 g, 0.34, 0.23 to 3195 g, −76.45 g·s, 357.04%, 678.23 g, 968.56 g, 0.72, 0.55, respectively.

Starch gels prepared from M8 showed the highest hardness, followed by those from X1, whereas those prepared from M2, M3 and M4 showed lower value (726 g, 529 g, 1008 g, Table S2). This was in line with the pasting and gelatinization temperatures, but it was opposite to the results of PV, FV BDV and setback. Differences in the hardness of the jackfruit starches were found, which implied that other factors affected this property because the amylose content was at the same level. The gel strength was correlated with the extent of particle swelling, according to previous studies^21,27^. In addition, it was indicated that hardness of rice starch was caused mainly by retrogradation of starch gels, which was associated with crystallization of amylose for a short time^23,26^. Starches with harder gels tended to have longer and a higher proportion of amylopectin chains, which might explain the present observation for M8. The starch with smallest particle size had the highest FV and setback viscosity, which could result in harder gelatinization, based on the results of Zhang *et al*.^9^. With the smallest particle size and a perfect amylopectin shoulder structure (Table S1 and Fig. 2A), X1 displayed the most perfect crystalline, as Table 4 and Fig. 4b show. This starch would have more consistent gelatinization after the retrogradation of amylose and amylopectin, which would lead to a harder gel.

The starch gels from the group containing M2, M3 and M4 exhibited a higher value for adhesiveness, followed by X1, whereas M8 starch gels had the lowest value (Table S2). Significant (p < 0.05) differences in adhesiveness were observed among the starch gels of jackfruits. This result was opposite to the hardness of starch in this study.

Springiness, chewiness, and resilience were highest for M8 and lowest for M3. M8 and M3 had higher gumminess and cohesiveness, but these were lower for X1 and M4 (Table S2).

These TPA results indicated that M8 had the most rigid gel network, whereas M2, M3 and M4 had the stickiest gels.

## Discussion

A key finding of the present study was that the most important intrinsic factors that determined the starch physicochemical properties were the molar mass of amylose (AM) and amylopectin (AP) and the fine structure of the same AP.

Significant positive relationships were observed between Mw and Mn, Rg of the amylose and amylopectin (r = 0.93, 0.91, 0.92, 0.91, p < 0.01). However, the Mw, Mn, and Rg of the amylose had a significant negative correlation (r = −0.93) with those of amylopectin. Similar result have also been reported in previous studies^17,18^. These results implied that the molecular size increased with the increasing molecular weight of amylose and amylopectin, or vice versa. However, the prevalent AP molecules were of increased molecular sizes, the amylose molecules were of decreased molecular sizes. Except for X1, the Mw of the amylose displayed a significant positive correlation with the proportion of very long amylopectin branch chains (DP 25–36, DP ≥ 37) and CL (r = 0.93, 0.94, 0.95, p < 0.01) but was correlated negatively with short average amylopectin branch chains (DP 6–12) (r = −0.95, p < 0.01). The Mw of the amylopectin displayed significant positive correlation with short average amylopectin branch chains (DP 6–12) (r = 0.75, p < 0.05) but was negatively correlated with the proportion of very long amylopectin branch chains (DP 25–36) and CL (r = −0.70, −0.71, p < 0.05). A higher proportion of amylopectin fractions (DP 26–36, ≥37) had significant positive relation with the average chain length (r = 0.95, 0.90, p < 0.01). These results indicated that a higher proportion of very short chains could form a more compact structure in larger AP but smaller amylose. In addition, a higher proportion of long chains were associated with a high average chain length.

Based on previous results^11,12,20^, because it had the highest proportion of DP 6–12, the smallest proportion of DP 25–36, DP ≥ 37, and the shortest chain length, the X1 starch was expected to have a higher peak viscosity, breakdown, final viscosity, setback, R, adhesiveness and lower pasting temperature, gelatinization temperature, hardness than the other starches. However, the opposite result was observed for X1. The PV and BDV results could be ascribed to the lower Mw of amylopectin (3.61 × 10^7^ g/mol), its smallest particle size (7.63 μm) and its Rg (94.6 nm). The FV and setback results were caused by the higher Mw of amylose (2.61 × 10^7^ g/mol). For the PV, BDV, FV and setback results from pasting properties, the molar mass (Mw) and molecular size (Rg) had a more significant effect than did the chain profile distribution for X1. Based on previous results^22,26^, with its perfect amylopectin shoulder structure, smallest amylopectin size (Rg) and highest proportion of short amylopectin branch chains, X1 can form the most perfect and smallest amount of crystalline structures compared to the other starch samples. As a result, X1 could break down the much less ordered double helices when it was gelatinized. It also had a lower enthalpy (ΔHg) and relative crystallinity than did the other starches. Based on the pasting and gelatinization temperatures, a perfect crystalline structure was found for X1, when Fig. 3 does not show a shoulder structure in the chromatogram, which could contribute to the very high pasting and gelatinization temperature. In addition, according to previous literature^9,14,17,28^, the higher Mw of amylose and the smaller amylopectin and starch particle size could lead to a higher pasting and gelatinization temperature, which might have implicated with the present observations for M1. For the hardness and adhesiveness results based on its textural properties, X1 had more consistent gelatinization after the retrogradation of amylose and amylopectin because of its smallest particle size and perfect amylopectin shoulder structure (Table S1 and Fig. 2A), which led to harder gelatinization. In addition, the higher Mw of amylose, lower Mw of amylopectin, smallest particle size and amylopectin Rg could also cause the formation of a smaller amount and harder gel. The molar mass, particle size and amylopectin Rg as well as the amylopectin structure, played more significant effect than did the chain profile distribution for the pasting temperature, gelatinization temperature and texture properties.

Many studies have reported on the relationship between all kinds of physicochemical properties, such as the proximate composition, size distribution, morphology, swelling power, solubility, and pasting, thermal, crystal, textural, digestion, and retrogradation properties^29–33^. In addition, the reasons for the changes in the physicochemical properties were ascribed to different amylose contents, particle sizes and starch components^29,30,34,35^. In this study, except for X1, the PV, BDV, FV, and setback had a significant negative correlation with the Mw of amylose and the proportion of very long amylopectin branch chains (DP 25–36, DP ≥ 37) and the CL (r = −0.94, −0.91, −0.97, −0.91, p < 0.01; r = −0.94, −0.91, −0.88, −0.89, p < 0.01; r = −0.86, −0.86, −0.87, −0.87, p < 0.01; r = −0.91, −0.91, −0.92, −0.92, p < 0.01) but had a significant positive correlation with the Mw of amylopectin and the proportion of short and medium average amylopectin branch chains (DP 6–12, DP 13–24). These results were also generally consistent with previous reports that Mn in AP was positively correlated with the peak viscosity of wheat and rice starch^10,36^. However, the opposite result was reported that the Mw of amylopectin was negatively correlated with the peak viscosity and setback of long-grain rice starches^37^. The discrepancy could be ascribed to the higher Mw of amylopectin (1.21–1.53 × 10^7^ g/mol), the proportion of DP 13–24 (52.1–53.1%), DP ≥ 37 (9.8–10.4%) and the lower amylose content (18.6–22.1%), DP 6–12 (23.8–24.4), 25–36 (13.2%) for the long-grain rice starches than the jackfruit starch used in this study. The Mw of AP was reported to be negatively correlated with the setback but not with the peak viscosity of cassava starches^38^. It was reported no correlations between Mw and the pasting properties of Taiwanese waxy rice starches^39^. These results for starches of different origins could be explained by the particle size distribution, morphology, average Mw of amylose and amylopectin, and branch chain-length distribution of amylopectin. The pasting temperature had an inverse relationship with the Mw of amylose and amylopectin, the proportion of short, medium, long amylopectin branch chains and the CL (r = 0.84, −0.88, −0.91, −0.74, 0.87, 0.90, 0.90, p < 0.05). This result agreed with previous results^10,37,38^. The PV had a significant correlation with BDV, FV, setback and pasting temperature (r = 0.96, 0.95, 0.98, –0.94, p < 0.01) based on the molecular structural properties of the jackfruit starches. In contrast, rice starch had a lower PV and BDV and tended to have a higher FV and setback. The shape of those AP molecules was probably closer to the AM molecules, which easily formed associations between themselves and significantly contributed to network formation, resulting in higher values of FV and SB during cooling^10^. In this study, the Mw of jackfruit amylose was lower than that of amylopectin, and the proportion of DP 25–36, ≥37 was higher than that of rice starch (5.86–6.46%, 1.21–1.48%), which might have implicated with the present observation. Except for X1, the temperature parameters (To, Tp and Tc) had a significant positive relationship with the Mw of amylose, proportion of very long amylopectin branch chains (DP 25–36, DP ≥ 37) and CL (r = 0.94, 0.89, 0.82, 0.87; r = 0.94, 0.84, 0.88, 0.88; r = 0.93, 0.76, 0.73, 0.84, p < 0.05) but showed a significant negative correlation with the Mw of amylopectin and proportion of short average amylopectin branch chains (DP 6–12) (r = −0.84, −0.85, −0.71, r = −0.85, −0.89, −0.79 and −0.70, −0.76, −0.72, p < 0.05). This generally agreed with previous results for sweet potato starch^40^, waxy maize starch^41^, barley starch^42^, amaranth starch^43^ and wheat starch^24^, which indicated that a higher proportion of short chains of DP 6–12 resulted in a lower gelatinization temperature and enthalpy, whereas a higher proportion of chains of DP 24–36, ≥37 was correlated with a higher gelatinization temperature. The enthalpy (ΔHg) and relative crystallinity had a significant correlation with short, long, very long amylopectin branch chains and CL (r = −0.96, 0.97, 0.88, 0.94 and r = −0.88, 0.86, 0.70, 0.79, p < 0.05). The thermal and crystal properties were in accordance with the pasting properties based on the molecular structure. This finding was in accordance with previous studies on amaranth starch^42^ and wheat starch^24^ but was not with cassava starch^38^. A possible explanation may be that cassava starch granules have a highly homogeneous and ordered structure with a larger granule size and higher entanglement of amylopectin with higher proportion of low molecular weight amylopectin than jackfruit starches do.

Except for X1, gel hardness had a significant positive correlation with the Mw of amylose and the proportion of very long amylopectin branch chains (DP 25–36, DP ≥ 37) and the CL (r = 0.85, 0.85, 0.87, 0.91, p < 0.01) but showed a significant negative correlation with the Mw of amylopectin and proportion of short average amylopectin branch chains (DP 6–12) (r = −0.89, −0.85, p < 0.05). The opposite relationship was observed between adhesiveness and the starch molecular structure. The textural properties were in accordance with the pasting, gelatinization and crystal properties, as the hardness was positively correlated with the pasting temperature, gelatinization temperature, ΔHg and relative crystallinity (r = 0.98, 0.88, 0.93, 0.76, 0.73, 0.70, p < 0.05) but was negatively correlated with the PV, BDV, FV, setback, R and adhesiveness (r = −0.96, −0.91, −0.95, −0.82, −0.91,−0.94, p < 0.01) on basis of molecular structure of jackfruit starch. This was similar to previous results for rice starch^44,45^.

## Conclusions

The starches of five varieties of an untraditional source, jackfruit (*Artocarpus heterophyllus Lam*.), planted in China, were isolated and characterized in terms of their molecular structure for the first time. A relationship was built between molecular structure and the physicochemical properties. The M8 had a higher amylose Mw, proportion of DP 25–36, DP ≥ 37, and CL, but M2, M3 and M4 had higher amylopectin Mw, and X1 had a higher proportion of DP 6–12 and a perfect amylopectin structure. A shoulder at DP 14–18 was observed for all amylopectin samples except X1. For a similar amylose content, the textural properties were in accordance with the pasting, gelatinization and crystal properties because the hardness was positively correlated with the pasting temperature, gelatinization temperature, ΔHg and relative crystallites (r = 0.98, 0.88, 0.93, 0.76, 0.73, 0.70, p < 0.05) but negatively correlated with the PV, BDV, FV, setback, R and adhesiveness (r = −0.96, −0.91, −0.95, −0.82, −0.91, −0.94, p < 0.01) on the basis of the molecular structure of jackfruit starch. These results demonstrated that the molecular structure of starch plays a role in determining their physicochemical properties. These results could be used as a reference to further analyse the starch structure and the relationships between the starch structure and the physicochemical properties in the future.

## Materials and Methods

### Materials

Five jackfruits cultivars were harvested in May 2016 from the garden of the Spice and Beverage Research Institute (Hainan, China), and the seeds were obtained from the bulbs. All five jackfruits cultivars came from plants of the same age, harvest stage, climatic and agronomic conditions. Four exotic jackfruit cultivars were *Artocarpus heterophyllus Lam. cv. Malaixiya No. 2* (M2), *Malaixiya No. 3* (M3), *Malaixiya No. 4* (M4), *Malaixiya No. 8* (M8). The last was the local cultivar *Xiangyinsuo 1 hao* (X1).

### Starch extraction

Starch was extracted using the method described by Zhang *et al*. with some modifications^9^. The jackfruit seeds were stored at −40 °C for 3 hours and then dried at 60 °C for 45 min. The arils and thin brown spermoderms could be easily removed manually. The remaining pieces of spermoderms were removed by washing several times with distilled water.

The seeds were mixed with triple distilled water and broken in a colloid mill for 10 min. The seed fibres were eliminated by passage through a 200 mesh sieve after homogenization. The residual mixture was centrifuged at 2000 rpm for 30 min and the supernatant was discarded. The precipitate was re-suspended in a 0.5 M solution of sodium thiosulfate (1:1, precipitate to solution) for 36 h, during which it was stirred at regular intervals. It was centrifuged at 3000 rpm for 15 min. The supernatant was removed, and upper brown sediment was manually scraped. The sediment was neutralized until the pH reached 7.0. The residual was further washed with 50% ethanol three times. The white starch cake was dried at 50 °C until the moisture content was less than 13 g/100 g. The starch was ground and passed through a 100 mesh sieve. The prepared starch was packed in an aluminium foil bag until further analysis.

### Separation of amylose and amylopectin

The amylose and amylopectin were separated by the method of Zhong *et al*. with some modifications^14^. The jackfruit seed starch was mixed with water to a concentration of 3% (w/v), then placed into a bath shaker (Bo Xun Industrial Co., Ltd. medical equipment factory, Shang Hai, China) until the temperature reached 100 °C, where it was held for 1 h with continuous stirring. The samples were centrifuged at 5000 g/min for 10 min using a centrifuge (An Ting Scientific Instrument Factory, Shang Hai, China). The supernatant was mixed with 1/2 volume of absolute ethanol. The mixture was centrifuged after standing for 12 hours. The precipitate was amylose. To remove the remaining amylose completely, the precipitate from the first centrifugation was heated, stirred, and centrifuged again. The new precipitate was mixed with methanol to a concentration of 80% (v/v), vortexed (IKA, Shang Hai, China) for 2 min, and centrifuged. The precipitate was amylopectin. The amylose and amylopectin were vacuum freeze-dried (Xin Zhi Bioscience Co., Inc., Ningbo, China) to produce the samples for testing.

### Determination of average particle size, range and amylose

The average particle size and range of starch in solution were determined with a Nicomp 380/ZLS Zeta potential/Particle sizer (PSS Nicomp, Santa Barbara, USA) based on dynamic light scattering (DLS). The solutions were diluted to a concentration of 0.5 mg/mL with deionized water, and all measurements were obtained at 25 °C. The amylose content of starches defatted with 85% methanol solution was quantified using methods from the literatures^11,22,44,46^.

### Molecular weight distribution of amylose and amylopectin

Amylose and amylopectin Mw, Mn, Rz, and polydispersity (ratio of weight-average and number-average molar mass, Mw/Mn) were determined by HPSEC-MALLS-RI systems^14^. The HPSEC-MALLS-RI consisted of a LC-20AB Pump (Shimadzu, Japan), a HP1050 injector, a Waters 2414 differential refractometer (Waters, USA), and a Dawn DSP-F Multi-angle Laser Light-scattering Detector (Wyatt Technology Corporation, USA). High-performance Liquid Chromatographic Columns (Styragel® HMW 6E DMF 250 and Styragel® HMW 6E DMF 1000 Waters, USA) were connected in series for the SEC analysis. DMSO/NaNO_3_ (50 mM) was used as the mobile phase at a flow rate of 0.6 mL/min. The mobile phase was degassed for 20 min using ultrasonic and filtered through a 0.45 μm PTFE filter. The temperatures of the RI and LS detectors were maintained at 40 °C and 55 °C, respectively. A dextran standard (T40, 2 mg/ml) was used to normalize the multi-angle photodiode detector in the MALLS.

The samples were dissolved in a DMSO solution (50 mM) (DMSO/NaNO_3_), and the concentration was brought to 2 mg/mL. The mixture was stirred for 20 h in a magnetic agitator at 90 °C. All solutions were filtered with a 0.45 μm PTFE filter before the MALLS measurement.

The Astra version 5.3.4 software (Wyatt Technology, Santa Barbara, CA, USA) was used for data acquisition and analysis. A second-order Berry method was used for curve fitting and to calculate the molecular weight, mean square radius of gyration and polydispersity index.

### Amylopectin fine structure analysis

The amylopectin fine structure of the jackfruit starches was determined by high performance anion-exchange chromatography with pulsed amperometric detection (HPAEC-PAD, Dionex ICS-5000, Dionex Corporation, Sunnyvale, CA, USA)^20,47^. The sample (40 mg) was mixed with 2 mL of acetic acid buffer (50 mM, pH 6.0) in a test tube with a plug and cooked in a boiling water bath with stirring for 30 min. After the mixture was cooled to 55 °C, 10 μL of pullulanase was added, and the mixture was stirred for 15 h in a magnetic agitator at 55 °C. The solution was placed in a boiling water bath for 20 min, and an aliquot of the mixture was centrifuged at 5000 g for 10 min. The supernatant was filtered through a 0.45 μm filter and diluted 10-fold before injection for analysis. The HPAEC system consisted of an ED50 electrochemical detector and the CarboPac PA200 column (3 × 250 mm, Dionex Corporation, Sunnyvale, CA, USA). The mobile phase was a gradient eluent with 150 mM NaOH and 500 mM sodium acetate in 150 mM NaOH, and the flow rate was set as 0.5 mL/min.

### Pasting properties

The pasting properties were studied with a Rapid Visco Analyzer (RVA super 4, Newport Scientific, Australia). An aliquot of 3.0 g of starch sample was mixed with 25.0 mL water and stirred in an RVA container initially at 960 r/min for 10 secs and then at 160 r/min for the remainder of the procedure. The temperature-time conditions included a heating step from 50 to 95 °C at 6 °C /min (after an equilibration time of 1 min at 50 °C), a holding phase at 95 °C for 5 min, a cooling step from 95 to 50 °C at 6 °C/min and a holding phase at 50 °C for 2 min. The parameters recorded were peak viscosity, breakdown, final viscosity, setback viscosity, and pasting temperature. All measurements were replicated three times.

### Thermal properties

The thermal properties were determined using a differential scanning calorimeter (DSC-Q2000 TA Instruments, USA), equipped with a thermal analysis data station (TA Instruments, Newcastle, DE)^26^. A aliquot of 2 mg sample (dry basis) was loaded into a 40 mL aluminium pan, and distilled water was added to achieve a starch-water suspension containing 70% water. The samples were hermetically sealed and allowed to stand for 24 h at room temperature before heating in the DSC. The sample pans were heated from 20 to 100 °C at a rate of 10 °C /min. Indium and an empty aluminium pan were used as reference to calibrate the DSC. The onset temperature (To), peak temperature (Tp), conclusion temperature (Tc) and gelatinization enthalpy (ΔH) were calculated by a Universal Analysis Program (TA Instruments). The analyses were performed in triplicate.

### Crystal structure analysis

The crystal structure of starch was determined with a Di System X-ray diffractometer (Bede XRD Di System, Durham, United Kingdom) equipped with a copper tube operating at 40 kV and 200 mA, producing CuKα radiation at a 0.154 nm wave length. Diffractograms were obtained by scanning from 4° to 40° (2θ) at a rate of 4°/min, a divergence slit width of 1°, a step size of 0.02°, a scatter slit width of 1° and a receiving slit width of 0.02 mm. The relative crystallinity of starches was calculated as the area ratio of the crystalline sharp peak over the total area using peak-fitting software (Origin-version 8.5, Microcal Inc., Northampton, MA, USA)^26^. All measurements were replicated three times.

### Texture profile analysis

The textural profile analysis (TPA) of starch samples was performed according to literature methods with some modifications^21,27^. Starch (17%, dwb) mixed with distilled water was cooked for 30 min in a boiling water bath with moderate mechanical agitation then cooled in a mold. The texture of the starch gel was determined using a texture analyser (TA. XT. plus, Texture Technologies Corp., UK) with a 50 kg load cell with a two-cycle compression. The analyser was linked to a computer that recorded the data via the Texture Expert Excede Version 1.0 software package (Stable Micro Systems Software). A P/0.5 R cylinder probe was used to carry out the TPA pattern with 40% strain, a pre-test speed of 1.0 mm/s, a test speed of 2 mm/s and a post-test speed of 10 mm/s. Hardness, adhesive force, springiness, gumminess, cohesiveness were determined, and the values reported were the mean of ten replications.

### Statistical analysis

The mean, standard deviations and significant differences between the values and the correlation between parameters were calculated using SPSS 12.0.1 (SPSS Inc., Chicago, IL, USA). Tests of significant differences between means were determined by Duncan’s multiple range tests at a significance level of 0.05. The data reported in all of the tables were the average of triplicate observations.

## Electronic supplementary material

Supplementary Tables
