# Supplementary material for: Molecular structure of starch isolated from jackfruit and its relationship with physicochemical properties
Source: Sci Rep. 2017 Oct 18;7:13423. doi: 10.1038/s41598-017-13435-8 (PMC5647410; doi:10.1038/s41598-017-13435-8)
Supplement: Supplementary file 1 — Supplementary Tables [file 41598_2017_13435_MOESM1_ESM.doc]

Molecular structure of starch isolated from jackfruit and its relationship with physicochemical properties

Yanjun Zhang ^a^, Yutong Zhang ^b^, Fei Xu ^a^, Gang Wu ^a^, Lehe Tan a [[1]](#footnote-2)*

a Spice and Beverage Research Institute, Chinese Academy of Tropical Agricultural Sciences, Wanning, 571533, Hainan, China

b College of Food Science, Bayi Agricultural University, Daqing, Heilongjiang 158308, China

Word count of text: 7082 words

Running title: Special properties of jackfruit cultivars starches for utilization

Table 1S Granule size and amylose content of jackfruit seed starches

| Cultivars | Diameter  range (mm) | Average  diameter (mm) | **Amylose [%, dry basis, db]** |
| --- | --- | --- | --- |
| **X1** | 0.41-30.77 | 7.63±0.98d | 32.56±0.32a |
| **M8** | 0.43-31.43 | 10.29±1.12c | 32.34±0.26a |
| **M4** | 0.49-33.48 | 10.68±1.15bc | 30.05±0.26a |
| **M3** | 0.46-46.95 | 12.46±1.18ab | 32.14±0.32a |
| **M2** | 0.56-32.58 | 11.54±1.22bc | 31.80±0.36a |

Values followed by the same letter in the same column are not significantly different (P<0.05).

Table 2S Texture profiles of seed starches isolated from five different jackfruits

| **Parameters** | **Hardness （g）** | **Adhesiveness (****g·s)** | **Springiness (%)** | **Gumminess (g)** | **Chewiness (g)** | **Resilience** | **Cohesiveness** |
| --- | --- | --- | --- | --- | --- | --- | --- |
| **X1** | 2500b | -129.68d | 201.60c | 309.68e | 667.54c | 0.56b | 0.33b |
| **M8** | 3195a | -152.63e | 357.04a | 678.23a | 968.56a | 0.72a | 0.38b |
| **M4** | 1008c | -93.85b | 252.46b | 468.98c | 869.32b | 0.51b | 0.23c |
| **M3** | 529e | -76.45a | 138.26d | 574.77b | 150.96d | 0.34c | 0.55a |
| **M2** | 726d | -107.41c | 140.28d | 367.27d | 164.46d | 0.38c | 0.49a |

Values followed by the same letter in the same column are not significantly different (P<0.05).

1. ^*^ Address for correspondence: Lehe Tan, Spice and Beverage Research Institute, Chinese Academy of Tropical Agricultural Sciences, CATAS, Wanning, Hainan, 571533, China. Fax: +86-898-6256-1083. E-Mail: tlh3687@163.com [↑](#footnote-ref-2)
